# (μ-Butane-1,2,3,4-tetra­carboxyl­ato)bis­[triaqua­(1,10-phenanthroline)nickel(II)] hexa­hydrate

**DOI:** 10.1107/S1600536810045137

**Published:** 2010-11-10

**Authors:** Hong-lin Zhu

**Affiliations:** a State Key Laboratory Base of Novel Functional Materials and Preparation Science, Center of Applied Solid State Chemistry Research, Ningbo University, Ningbo, Zhejiang 315211, People’s Republic of China

## Abstract

The asymmetric unit of the title compound, [Ni_2_(C_8_H_6_O_8_)(C_12_H_8_N_2_)_2_(H_2_O)_6_]·6H_2_O, contains a half of the centrosymmetric dinuclear complex mol­ecule and three uncoordinated water mol­ecules. In the dinuclear mol­ecule, two Ni^II^ cations are bridged by the butane-1,2,3,4-tetra­carboxyl­ate (BTC^4−^) anion. Each Ni^II^ atom is coordinated by two N atoms from the 1,10-phenanthroline ligand, one O atom from the BTC^4−^ anion and three aqua ligands in a distorted octa­hedral geometry. Inter­molecuar O—H⋯O hydrogen bonds and π–π stacking inter­ations [centroid–centroid distances = 3.646 (2), 3.781 (2) and 3.642 (2) Å] consolidate the crystal packing.

## Related literature

For related structures, see: Chen *et al.* (2008 ▶); Ghosh *et al.* (2004 ▶); Fabelo *et al.* (2008 ▶); Zhu & Zheng (2010 ▶).
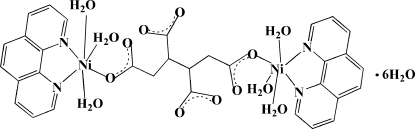


## Experimental

### 

#### Crystal data

[Ni_2_(C_8_H_6_O_8_)(C_12_H_8_N_2_)_2_(H_2_O)_6_]·6H_2_O
*M* *_r_* = 924.15
Triclinic, 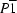

*a* = 9.0382 (18) Å
*b* = 9.5342 (19) Å
*c* = 12.253 (3) Å
α = 91.90 (3)°
β = 97.14 (3)°
γ = 111.54 (3)°
*V* = 971.0 (3) Å^3^
*Z* = 1
Mo *K*α radiation
μ = 1.06 mm^−1^
*T* = 293 K
0.43 × 0.39 × 0.32 mm

#### Data collection

Rigaku R-AXIS RAPID diffractometer
Absorption correction: multi-scan (*ABSCOR*; Higashi, 1995 ▶) *T*_min_ = 0.641, *T*_max_ = 0.713
7940 measured reflections
4195 independent reflections
3499 reflections with *I* > 2σ(*I*)
*R*_int_ = 0.022

#### Refinement

*R*[*F* ^2^ > 2σ(*F* ^2^)] = 0.034
*wR*(*F* ^2^) = 0.108
*S* = 1.17
4195 reflections
262 parameters
H-atom parameters constrained
Δρ_max_ = 0.74 e Å^−3^
Δρ_min_ = −0.48 e Å^−3^

### 

Data collection: *RAPID-AUTO* (Rigaku, 1998 ▶); cell refinement: *RAPID-AUTO*; data reduction: *CrystalStructure* (Rigaku/MSC, 2004 ▶); program(s) used to solve structure: *SHELXS97* (Sheldrick, 2008 ▶); program(s) used to refine structure: *SHELXL97* (Sheldrick, 2008 ▶); molecular graphics: *SHELXTL* (Sheldrick, 2008 ▶); software used to prepare material for publication: *SHELXL97*.

## Supplementary Material

Crystal structure: contains datablocks global, I. DOI: 10.1107/S1600536810045137/cv2791sup1.cif

Structure factors: contains datablocks I. DOI: 10.1107/S1600536810045137/cv2791Isup2.hkl

Additional supplementary materials:  crystallographic information; 3D view; checkCIF report

## Figures and Tables

**Table 1:** Hydrogen-bond geometry (Å, °)

*D*—H⋯*A*	*D*—H	H⋯*A*	*D*⋯*A*	*D*—H⋯*A*
O5—H5*B*⋯O10^i^	0.87	1.95	2.826 (4)	175
O5—H5*C*⋯O9^ii^	0.84	1.95	2.745 (3)	157
O6—H6*B*⋯O2	0.80	1.91	2.698 (3)	165
O6—H6*C*⋯O8	0.82	1.92	2.741 (4)	175
O7—H7*B*⋯O4	0.84	2.21	3.033 (3)	168
O7—H7*C*⋯O4^iii^	0.85	1.87	2.700 (3)	163
O8—H8*A*⋯O3^iv^	0.83	1.97	2.798 (3)	179
O8—H8*B*⋯O10^i^	0.86	2.03	2.891 (4)	179
O9—H9*B*⋯O2^i^	0.79	1.93	2.721 (3)	177
O9—H9*C*⋯O3^v^	0.84	2.11	2.867 (4)	149
O10—H10*B*⋯O4^vi^	0.82	1.93	2.743 (3)	166
O10—H10*C*⋯O9^vii^	0.87	2.06	2.886 (3)	159

Symmetry codes: (i) 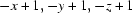; (ii) 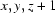; (iii) 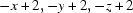; (iv) 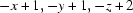; (v) 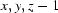; (vi) 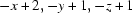; (vii) 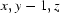.

**Fig. 1.**
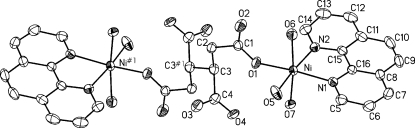
View of the title compound showing the atomic numbering and 45% probability dispalcement ellipsoids [symmetry code: (#1) -x + 2, -y + 1, -z + 2]. The lattice water molecules and H-atoms omitted for clarity

**Fig. 2.**
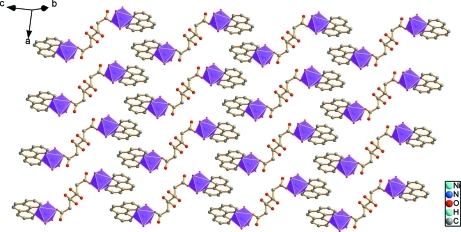
Supramolecular assembly of two-dimensional layer through π–π stacking interactions between the phen ligands.

## supplementary crystallographic information

### Comment

So far, the multi-carboxylate ligands such as benzene-, pyridine- and aliphatic carboxylic acid are widely used to construct the coordination polymers with interesting structures (Chen *et al.*, 2008; Ghosh *et al.*, 2004; Fabelo *et al.*, 2008; Zhu *et al.*, 2010). Among the various polycarboxylate ligands, butane-1,2,3,4-tetracarboxylic acid has been to be a good candidate due to it has four COOH groups which can be fully or patially deprotonated and its various bridging abilities and strong coordination tendency with transition metals to form versatile coordination polymers or supramolecular architecture. In this paper, we report the synthesis and crystal structure of the title compound (Fig. 1).

Within the asymmetric unit of the title compound exists one Ni^II^ cation, one 1,10-phenanthroline (phen) ligand, half a butane-1,2,3,4-tetracarboxylate anion (BTC^4-^), three aqua ligands and three lattice water. The Ni atoms are each coordinated by two N atoms from one phen ligand, one oxygen atoms from one BTC^4-^ anion and three aqua ligands to complete an octahedral NiN_2_O_4_ chromophore. The Ni–N/O distances lie in the range 2.017 (2)–2.091 (2) Å, and the *trans*- and *cis* bond angles fall in the ranges 80.2 (1)–97.7 (1)° and 168.9 (1)- 172.6 (1)°, respectively. The [Ni(H_2_O)_3_(phen)] moieties are pairwise bridged by the bis-monodentate BTC ligand to generate centrosymmetric dinuclear complex molecules [Ni_2_(H_2_O)_6_(phen)_2_(BTC)], which are arranged in pairs and the interdigitatal distance for phen ligands of the adjacent dinuclear molecules fall in the regions 3.642 (2) Å—3.781 (2) Å, which indicates that the complex molecules are assembled into two-dimensional supramolecular layers parallel to (011) by π–π stacking interactions (Figure 2). Due to the aqua ligands donate hydrogen atoms to the carboxylate oxygen atoms to form interlayers hydrogen bonds, the two-dimensional layers are assembled into a three-dimensional supramolecular architecture.

### Experimental

All chemicals were obtained from commerical sources and were used as obtained. 1.0 ml (1.0 *M*) Na_2_CO_3_ was added to an aqueous solution of 0.2351 g (1.0 mmol) NiCl_2_.6H_2_O in 8 ml H_2_O to yield green precipitate, which was separated by centrifugation and washed with distilled water for 5 times. The gathered precipitate was then transferred into a mixture solutions of methanol and water (1:1 V/V, 20 ml). Then 0.1163 g (0.5 mmol) 1,2,3,4-butanetetracarboxylic acid and 0.1015 g (0.5 mmol) 1,10-phenanthroline monohydrate were added successively to the mixture solutions, which quickly produced green suspension. The resulting mixture was continued to stir for *ca* 30 min and then filtered green precipitate. The filtrate was allowed to stand at room temperature and slow evaporation for one month afforded green block-like crystals.

### Refinement

H atoms bonded to C atoms were palced in geometrically calculated position and were refined using a riding model, with *U*_iso_(H) = 1.2 *U*_eq_(C). H atoms attached to O atoms were found in a difference Fourier synthesis and were refined using a riding model, with the O—H distances fixed as initially found and with *U*_iso_(H) values set at 1.2 *U*_eq_(O).

### Crystal data

**Table:**

[Ni_2_(C_8_H_6_O_8_)(C_12_H_8_N_2_)_2_(H_2_O)_6_]·6H_2_O	*Z* = 1
*M**_r_* = 924.15	*F*(000) = 482
Triclinic, *P*1	*D*_x_ = 1.580 Mg m^−^^3^
Hall symbol: -P 1	Mo *K*α radiation, λ = 0.71073 Å
*a* = 9.0382 (18) Å	Cell parameters from 6619 reflections
*b* = 9.5342 (19) Å	θ = 3.1–27.4°
*c* = 12.253 (3) Å	µ = 1.06 mm^−^^1^
α = 91.90 (3)°	*T* = 293 K
β = 97.14 (3)°	Block, green
γ = 111.54 (3)°	0.43 × 0.39 × 0.32 mm
*V* = 971.0 (3) Å^3^	

### Data collection

**Table:**

Rigaku R-AXIS RAPID diffractometer	4195 independent reflections
Radiation source: fine-focus sealed tube	3499 reflections with *I* > 2σ(*I*)
graphite	*R*_int_ = 0.022
Detector resolution: 0 pixels mm^-1^	θ_max_ = 27.4°, θ_min_ = 3.1°
ω scans	*h* = −11→11
Absorption correction: multi-scan (*ABSCOR*; Higashi, 1995)	*k* = −12→10
*T*_min_ = 0.641, *T*_max_ = 0.713	*l* = −15→15
7940 measured reflections	

### Refinement

**Table:**

Refinement on *F*^2^	Primary atom site location: structure-invariant direct methods
Least-squares matrix: full	Secondary atom site location: difference Fourier map
*R*[*F*^2^ > 2σ(*F*^2^)] = 0.034	Hydrogen site location: inferred from neighbouring sites
*wR*(*F*^2^) = 0.108	H-atom parameters constrained
*S* = 1.17	*w* = 1/[σ^2^(*F*_o_^2^) + (0.0364*P*)^2^ + 1.1101*P*] where *P* = (*F*_o_^2^ + 2*F*_c_^2^)/3
4195 reflections	(Δ/σ)_max_ = 0.001
262 parameters	Δρ_max_ = 0.74 e Å^−^^3^
0 restraints	Δρ_min_ = −0.48 e Å^−^^3^

### Special details

**Table:**

Geometry. All e.s.d.'s (except the e.s.d. in the dihedral angle between two l.s. planes) are estimated using the full covariance matrix. The cell e.s.d.'s are taken into account individually in the estimation of e.s.d.'s in distances, angles and torsion angles; correlations between e.s.d.'s in cell parameters are only used when they are defined by crystal symmetry. An approximate (isotropic) treatment of cell e.s.d.'s is used for estimating e.s.d.'s involving l.s. planes.
Refinement. Refinement of *F*^2^ against ALL reflections. The weighted *R*-factor *wR* and goodness of fit *S* are based on *F*^2^, conventional *R*-factors *R* are based on *F*, with *F* set to zero for negative *F*^2^. The threshold expression of *F*^2^ > σ(*F*^2^) is used only for calculating *R*-factors(gt) *etc*. and is not relevant to the choice of reflections for refinement. *R*-factors based on *F*^2^ are statistically about twice as large as those based on *F*, and *R*- factors based on ALL data will be even larger.

### Fractional atomic coordinates and isotropic or equivalent isotropic displacement parameters (Å^2^)

**Table:**

	*x*	*y*	*z*	*U*_iso_*/*U*_eq_	
Ni	0.68724 (4)	0.80076 (4)	0.76505 (3)	0.02477 (12)	
N1	0.6678 (3)	0.9786 (3)	0.67867 (18)	0.0276 (5)	
N2	0.7744 (3)	0.7621 (3)	0.62136 (19)	0.0277 (5)	
O5	0.5825 (3)	0.8507 (3)	0.89489 (17)	0.0423 (5)	
H5B	0.4869	0.8552	0.8872	0.051*	
H5C	0.6378	0.8539	0.9561	0.051*	
O6	0.4684 (2)	0.6335 (2)	0.69310 (17)	0.0341 (5)	
H6B	0.4833	0.5630	0.7195	0.041*	
H6C	0.3783	0.6165	0.7092	0.041*	
O7	0.9146 (2)	0.9445 (2)	0.84282 (16)	0.0293 (4)	
H7B	0.9588	0.9008	0.8865	0.035*	
H7C	0.9255	1.0310	0.8718	0.035*	
O1	0.7521 (2)	0.6486 (2)	0.84846 (17)	0.0362 (5)	
O2	0.5423 (2)	0.4320 (2)	0.8157 (2)	0.0425 (5)	
C1	0.6809 (3)	0.5127 (3)	0.8629 (2)	0.0271 (6)	
C2	0.7714 (3)	0.4396 (3)	0.9396 (2)	0.0290 (6)	
H2A	0.7638	0.3446	0.9035	0.035*	
H2B	0.7215	0.4176	1.0059	0.035*	
C3	0.9509 (3)	0.5431 (3)	0.9717 (2)	0.0295 (6)	
H3A	0.9948	0.5802	0.9044	0.035*	
C4	0.9715 (3)	0.6820 (3)	1.0517 (2)	0.0309 (6)	
O3	0.9006 (3)	0.6585 (2)	1.13534 (18)	0.0407 (5)	
O4	1.0589 (3)	0.8098 (2)	1.02824 (17)	0.0347 (5)	
C5	0.6195 (4)	1.0876 (4)	0.7097 (3)	0.0359 (7)	
H5A	0.5862	1.0870	0.7786	0.043*	
C6	0.6166 (4)	1.2032 (4)	0.6433 (3)	0.0434 (8)	
H6A	0.5815	1.2773	0.6678	0.052*	
C7	0.6657 (4)	1.2063 (4)	0.5420 (3)	0.0415 (8)	
H7A	0.6635	1.2821	0.4967	0.050*	
C8	0.7197 (4)	1.0935 (4)	0.5066 (2)	0.0347 (6)	
C9	0.7752 (4)	1.0884 (4)	0.4024 (3)	0.0431 (8)	
H9A	0.7787	1.1638	0.3554	0.052*	
C10	0.8223 (4)	0.9765 (4)	0.3714 (2)	0.0427 (8)	
H10A	0.8570	0.9755	0.3032	0.051*	
C11	0.8201 (4)	0.8591 (4)	0.4419 (2)	0.0346 (7)	
C12	0.8641 (4)	0.7363 (4)	0.4137 (3)	0.0432 (8)	
H12A	0.8931	0.7258	0.3446	0.052*	
C13	0.8637 (4)	0.6342 (4)	0.4880 (3)	0.0460 (8)	
H13A	0.8919	0.5528	0.4698	0.055*	
C14	0.8207 (4)	0.6510 (3)	0.5925 (3)	0.0361 (7)	
H14A	0.8248	0.5817	0.6433	0.043*	
C15	0.7721 (3)	0.8636 (3)	0.5468 (2)	0.0278 (6)	
C16	0.7193 (3)	0.9821 (3)	0.5787 (2)	0.0265 (6)	
O8	0.1691 (3)	0.5950 (3)	0.74263 (19)	0.0460 (6)	
H8A	0.1488	0.5207	0.7791	0.055*	
H8B	0.2014	0.6772	0.7847	0.055*	
O9	0.6790 (3)	0.8105 (2)	0.10898 (17)	0.0368 (5)	
H9B	0.6128	0.7421	0.1314	0.044*	
H9C	0.7676	0.7992	0.1111	0.044*	
O10	0.7218 (3)	0.1261 (3)	0.11685 (18)	0.0414 (5)	
H10B	0.7796	0.1543	0.0685	0.050*	
H10C	0.6930	0.0308	0.1270	0.050*	

### Atomic displacement parameters (Å^2^)

**Table:**

	*U*^11^	*U*^22^	*U*^33^	*U*^12^	*U*^13^	*U*^23^
Ni	0.02751 (19)	0.0260 (2)	0.02009 (18)	0.00896 (15)	0.00360 (12)	0.00473 (13)
N1	0.0332 (12)	0.0274 (12)	0.0232 (11)	0.0130 (10)	0.0028 (9)	0.0010 (9)
N2	0.0303 (12)	0.0245 (12)	0.0271 (11)	0.0088 (10)	0.0050 (9)	0.0010 (9)
O5	0.0404 (12)	0.0683 (16)	0.0246 (10)	0.0258 (12)	0.0092 (9)	0.0079 (10)
O6	0.0273 (10)	0.0366 (12)	0.0340 (11)	0.0079 (9)	0.0003 (8)	0.0058 (9)
O7	0.0298 (10)	0.0219 (9)	0.0316 (10)	0.0056 (8)	0.0010 (8)	−0.0010 (8)
O1	0.0333 (11)	0.0268 (11)	0.0411 (12)	0.0053 (9)	−0.0048 (9)	0.0126 (9)
O2	0.0284 (11)	0.0325 (12)	0.0570 (14)	0.0039 (10)	−0.0069 (10)	0.0115 (10)
C1	0.0299 (14)	0.0277 (14)	0.0254 (13)	0.0121 (12)	0.0061 (10)	0.0038 (11)
C2	0.0279 (14)	0.0259 (14)	0.0317 (14)	0.0088 (12)	0.0014 (11)	0.0065 (11)
C3	0.0286 (14)	0.0297 (15)	0.0299 (14)	0.0101 (12)	0.0051 (11)	0.0063 (11)
C4	0.0323 (14)	0.0278 (15)	0.0372 (15)	0.0141 (13)	0.0122 (12)	0.0042 (12)
O3	0.0549 (14)	0.0318 (11)	0.0429 (12)	0.0187 (11)	0.0256 (10)	0.0068 (9)
O4	0.0410 (12)	0.0183 (10)	0.0408 (12)	0.0043 (9)	0.0133 (9)	−0.0007 (8)
C5	0.0408 (17)	0.0344 (16)	0.0326 (15)	0.0155 (14)	0.0025 (12)	−0.0020 (12)
C6	0.052 (2)	0.0318 (17)	0.0468 (19)	0.0198 (16)	−0.0031 (15)	−0.0022 (14)
C7	0.0484 (19)	0.0286 (16)	0.0433 (18)	0.0126 (15)	−0.0047 (14)	0.0088 (13)
C8	0.0374 (16)	0.0332 (16)	0.0275 (14)	0.0081 (13)	−0.0024 (12)	0.0074 (12)
C9	0.0454 (18)	0.048 (2)	0.0273 (15)	0.0073 (16)	0.0024 (13)	0.0158 (14)
C10	0.0431 (18)	0.056 (2)	0.0204 (14)	0.0082 (16)	0.0061 (12)	0.0070 (13)
C11	0.0298 (14)	0.0429 (17)	0.0242 (14)	0.0065 (13)	0.0027 (11)	−0.0031 (12)
C12	0.0417 (17)	0.052 (2)	0.0313 (16)	0.0119 (16)	0.0088 (13)	−0.0114 (15)
C13	0.0459 (19)	0.047 (2)	0.048 (2)	0.0214 (17)	0.0077 (15)	−0.0147 (16)
C14	0.0389 (16)	0.0293 (15)	0.0414 (17)	0.0140 (13)	0.0071 (13)	0.0003 (13)
C15	0.0273 (13)	0.0307 (15)	0.0211 (12)	0.0067 (12)	0.0023 (10)	−0.0006 (10)
C16	0.0271 (13)	0.0255 (14)	0.0222 (13)	0.0051 (11)	0.0008 (10)	0.0021 (10)
O8	0.0597 (15)	0.0415 (13)	0.0427 (13)	0.0203 (12)	0.0224 (11)	0.0110 (10)
O9	0.0392 (12)	0.0368 (12)	0.0362 (11)	0.0151 (10)	0.0084 (9)	0.0080 (9)
O10	0.0485 (13)	0.0418 (13)	0.0422 (12)	0.0214 (11)	0.0208 (10)	0.0104 (10)

### Geometric parameters (Å, °)

**Table:**

Ni—O1	2.017 (2)	C5—C6	1.397 (5)
Ni—O5	2.076 (2)	C5—H5A	0.9300
Ni—N1	2.078 (2)	C6—C7	1.367 (5)
Ni—O7	2.090 (2)	C6—H6A	0.9300
Ni—N2	2.091 (2)	C7—C8	1.410 (5)
Ni—O6	2.095 (2)	C7—H7A	0.9300
N1—C5	1.328 (4)	C8—C16	1.403 (4)
N1—C16	1.361 (3)	C8—C9	1.435 (4)
N2—C14	1.326 (4)	C9—C10	1.346 (5)
N2—C15	1.356 (4)	C9—H9A	0.9300
O5—H5B	0.8747	C10—C11	1.433 (5)
O5—H5C	0.8409	C10—H10A	0.9300
O6—H6B	0.8032	C11—C15	1.411 (4)
O6—H6C	0.8211	C11—C12	1.413 (5)
O7—H7B	0.8391	C12—C13	1.354 (5)
O7—H7C	0.8530	C12—H12A	0.9300
O1—C1	1.247 (3)	C13—C14	1.405 (5)
O2—C1	1.256 (3)	C13—H13A	0.9300
C1—C2	1.519 (4)	C14—H14A	0.9300
C2—C3	1.549 (4)	C15—C16	1.439 (4)
C2—H2A	0.9700	O8—H8A	0.8255
C2—H2B	0.9700	O8—H8B	0.8567
C3—C3^i^	1.537 (5)	O9—H9B	0.7902
C3—C4	1.561 (4)	O9—H9C	0.8441
C3—H3A	0.9800	O10—H10B	0.8251
C4—O4	1.251 (4)	O10—H10C	0.8664
C4—O3	1.259 (4)		
			
Cg1···Cg1^ii^	3.646 (2)	Cg2···Cg3^iii^	3.642 (2)
Cg1···Cg3^ii^	3.781 (2)		
			
O1—Ni—O5	92.22 (10)	C2—C3—H3A	108.5
O1—Ni—N1	168.90 (9)	C4—C3—H3A	108.5
O5—Ni—N1	93.36 (9)	O4—C4—O3	124.4 (3)
O1—Ni—O7	81.02 (8)	O4—C4—C3	117.3 (2)
O5—Ni—O7	90.53 (9)	O3—C4—C3	118.3 (3)
N1—Ni—O7	89.35 (9)	N1—C5—C6	122.8 (3)
O1—Ni—N2	94.72 (10)	N1—C5—H5A	118.6
O5—Ni—N2	172.60 (9)	C6—C5—H5A	118.6
N1—Ni—N2	80.24 (9)	C7—C6—C5	119.4 (3)
O7—Ni—N2	93.08 (9)	C7—C6—H6A	120.3
O1—Ni—O6	91.78 (9)	C5—C6—H6A	120.3
O5—Ni—O6	91.48 (9)	C6—C7—C8	119.4 (3)
N1—Ni—O6	97.65 (9)	C6—C7—H7A	120.3
O7—Ni—O6	172.59 (8)	C8—C7—H7A	120.3
N2—Ni—O6	85.75 (9)	C16—C8—C7	117.4 (3)
C5—N1—C16	118.2 (3)	C16—C8—C9	119.2 (3)
C5—N1—Ni	129.1 (2)	C7—C8—C9	123.4 (3)
C16—N1—Ni	112.61 (18)	C10—C9—C8	121.2 (3)
C14—N2—C15	117.9 (3)	C10—C9—H9A	119.4
C14—N2—Ni	129.5 (2)	C8—C9—H9A	119.4
C15—N2—Ni	112.43 (18)	C9—C10—C11	121.1 (3)
Ni—O5—H5B	123.9	C9—C10—H10A	119.4
Ni—O5—H5C	111.3	C11—C10—H10A	119.4
H5B—O5—H5C	124.2	C15—C11—C12	116.6 (3)
Ni—O6—H6B	96.9	C15—C11—C10	119.3 (3)
Ni—O6—H6C	128.3	C12—C11—C10	124.2 (3)
H6B—O6—H6C	98.2	C13—C12—C11	119.6 (3)
Ni—O7—H7B	112.7	C13—C12—H12A	120.2
Ni—O7—H7C	118.6	C11—C12—H12A	120.2
H7B—O7—H7C	110.8	C12—C13—C14	120.0 (3)
C1—O1—Ni	133.77 (19)	C12—C13—H13A	120.0
O1—C1—O2	124.4 (3)	C14—C13—H13A	120.0
O1—C1—C2	117.3 (2)	N2—C14—C13	122.4 (3)
O2—C1—C2	118.3 (3)	N2—C14—H14A	118.8
C1—C2—C3	111.9 (2)	C13—C14—H14A	118.8
C1—C2—H2A	109.2	N2—C15—C11	123.4 (3)
C3—C2—H2A	109.2	N2—C15—C16	117.2 (2)
C1—C2—H2B	109.2	C11—C15—C16	119.3 (3)
C3—C2—H2B	109.2	N1—C16—C8	122.7 (3)
H2A—C2—H2B	107.9	N1—C16—C15	117.4 (2)
C3^i^—C3—C2	111.6 (3)	C8—C16—C15	119.9 (3)
C3^i^—C3—C4	108.1 (3)	H8A—O8—H8B	111.0
C2—C3—C4	111.4 (2)	H9B—O9—H9C	112.7
C3^i^—C3—H3A	108.5	H10B—O10—H10C	114.8

Symmetry codes: (i) −*x*+2, −*y*+1, −*z*+2; (ii) −*x*+1, −*y*+2, −*z*+1; (iii) −*x*+2, −*y*+2, −*z*+1.

### Hydrogen-bond geometry (Å, °)

**Table:**

*D*—H···*A*	*D*—H	H···*A*	*D*···*A*	*D*—H···*A*
O5—H5B···O10^iv^	0.87	1.95	2.826 (4)	175
O5—H5C···O9^v^	0.84	1.95	2.745 (3)	157
O6—H6B···O2	0.80	1.91	2.698 (3)	165
O6—H6C···O8	0.82	1.92	2.741 (4)	175
O7—H7B···O4	0.84	2.21	3.033 (3)	168
O7—H7C···O4^vi^	0.85	1.87	2.700 (3)	163
O8—H8A···O3^vii^	0.83	1.97	2.798 (3)	179
O8—H8B···O10^iv^	0.86	2.03	2.891 (4)	179
O9—H9B···O2^iv^	0.79	1.93	2.721 (3)	177
O9—H9C···O3^viii^	0.84	2.11	2.867 (4)	149
O10—H10B···O4^ix^	0.82	1.93	2.743 (3)	166
O10—H10C···O9^x^	0.87	2.06	2.886 (3)	159

Symmetry codes: (iv) −*x*+1, −*y*+1, −*z*+1; (v) *x*, *y*, *z*+1;  (vi) −*x*+2, −*y*+2, −*z*+2; (vii) −*x*+1, −*y*+1, −*z*+2; (viii) *x*, *y*, *z*−1; (ix) −*x*+2, −*y*+1, −*z*+1; (x) *x*, *y*−1, *z*.
